# Faecal adrenal and thyroid hormones as markers of cold stress in Florida manatees (*Trichechus manatus latirostris*)

**DOI:** 10.1093/conphys/coag083

**Published:** 2026-11-12

**Authors:** Laetitia I Smoll, Elizabeth A Burgess, Tamara Keeley, Robert K Bonde, Martine de Wit, Janet M Lanyon

**Affiliations:** School of the Environment, Steele Building (Building 3), The University of Queensland, St. Lucia, Queensland 4072, Australia; Anderson Cabot Center for Ocean Life, New England Aquarium, Central Wharf, Boston, MA 02110, USA; School of Agriculture and Food Sustainability, The University of Queensland, 5391 Warrego Hwy, 5391 Warrego Hwy, Gatton, Queensland 4343, Australia; Clearwater Marine Aquarium Research Institute, 249 Windward Passage, 249 Windward Passage, Clearwater, FL 33767, USA; Marine Mammal Pathobiology Lab, Marine Mammal Pathobiology Lab, Fish and Wildlife Research Institute, Florida Fish and Wildlife Conservation Commission, 3700 54th Ave S, 3700 54th Ave S, St. Petersburg, FL 33701, USA; School of the Environment, Steele Building (Building 3), The University of Queensland, St. Lucia, Queensland 4072, Australia

**Keywords:** Aldosterone, cold stress syndrome (CSS), cortisol, endocrinology, faecal hormones, manatee, marine mammal, sirenian, thyroid hormone, watercraft collision

## Abstract

Florida manatees (*Trichechus manatus latirostris*) are susceptible to cold stress syndrome (CSS), a chronic, sometimes lethal, multisystem disease associated with skin lesions, emaciation, dehydration and secondary infections. To investigate endocrine changes associated with CSS, we compared faecal adrenal (cortisol and aldosterone) and thyroid hormone [total triiodothyronine (T3) and thyroxine (T4)] metabolite concentrations among manatees that died due to CSS, presumed healthy manatees that died acutely from watercraft collision injuries and live, apparently healthy wild manatees. Faecal T3 showed the strongest and most consistent association with health status, with CSS manatees exhibiting concentrations approximately three times higher than healthy animals. Elevated T3 concentrations may reflect increased thyroid hormone activity associated with thermoregulatory demands, nutritional stress or both. Faecal T4 was also elevated in CSS manatees, though this result was sensitive to the exclusion of calves and is complicated by strong cross-reactivity of the T4 assay with reverse T3 (rT3). Faecal cortisol concentrations were more strongly associated with size class than health status, with calves showing higher concentrations than adults regardless of group, potentially reflecting developmental and life-history factors including weaning and hypothalamic–pituitary–adrenal axis maturation. Faecal aldosterone showed an overall marginal effect of health status that was not maintained in pairwise comparisons or sensitivity analyses. Faecal hormone concentrations in manatees that died from acute watercraft injuries did not differ significantly from those of live, apparently healthy animals, providing some support for the interpretation that the differences in CSS manatees reflect ante-mortem physiological state rather than post-mortem sampling effects. Multivariate hormone profiles differed among health groups in the full dataset, though these patterns were strongly influenced by the disproportionate representation of immature animals in the CSS group. Together, these findings identify faecal T3 as a promising non-invasive indicator of CSS and improve understanding of endocrine responses to cold stress in manatees.

## Abbreviations

AIC, Akaike's information criterion
CFR, Code of Federal Regulations
CI, confidence interval
CITES, Convention on International Trade in Endangered Species of Wild Fauna and Flora
CRNWR, Crystal River National Wildlife Refuge
CSS, cold stress syndrome
df, degrees of freedom
fA, faecal aldosterone metabolites
fC, faecal cortisol metabolites
fT3, faecal triiodothyronine metabolites
fT4, faecal thyroxine metabolites
FWC, Florida Fish and Wildlife Conservation Commission
GLM, generalized linear model
HPA, hypothalamic–pituitary–adrenal
IPCC, Intergovernmental Panel on Climate Change
LC–MS/ MS, liquid chromatography–tandem mass spectrometry
MANOVA, multivariate analysis of variance
NOAA, National Oceanic and Atmospheric Administration
PC1, PC2, first and second principal components
PCA, principal component analysis
RAAS, renin–angiotensin–aldosterone system
rT3, reverse triiodothyronine
SD, standard deviation
T3, triiodothyronine
T4, thyroxine
UME, unusual mortality event
USGS, US Geological Survey
WC, watercraft collision

## Introduction

The Florida manatee (*Trichechus manatus latirostris*), a sub-species of the West Indian manatee, inhabits the heavily developed coastal and riverine systems of Florida and is consequently exposed to numerous human-induced stressors, including habitat degradation, entanglement in fishing gear and boat strikes, which can cause debilitation or death (Marsh *et al.,* 2011; Reinert *et al.,* 2017; Runge *et al.,* 2017). In addition to anthropogenic threats, manatees face many environmental disturbances such as red tide (algal bloom) events (Reep & Bonde, 2021) and food scarcity. Mortality numbers of Florida manatees tend to increase during winter because their low metabolic rates and thin fat layer (Irvine, 1983; Erdsack *et al.,* 2018) make them susceptible to cold stress syndrome (CSS) when temperatures drop below 20°C (Bossart *et al.,* 2002). CSS presents with a suite of chronic disease signs including peripheral skin lesions, emaciation, immune suppression, myocardial degeneration and inflammation of the intestines (Bossart *et al.,* 2002; Martony *et al.,* 2019). Due to their inability to withstand prolonged cold temperatures, Florida manatees rely primarily on behavioural thermoregulation, seeking out warm-water refuges (including natural springs and power plant effluents) to meet their thermal requirements in winter (Stith *et al.,* 2011; Laist *et al.,* 2013). From these wintering grounds, manatees commute to feeding grounds, travelling up to 40 km to feed on marine seagrass beds or to areas with other aquatic vegetation (Deutsch *et al.,* 2022). Coupled with the predicted increase in extreme weather events due to climate change (Vavrus *et al.,* 2006; Takahashi *et al.,* 2011), the planned closure of some coastal power plants and the phasing out of warm discharges from others over the next 30 years, there are serious concerns for the welfare and long-term survival of Florida manatees. Strategies are being developed to maintain a network of sustainable warm water refugia to ensure the recovery and persistence of the manatee in Florida waters (Valade *et al.,* 2020; Reep & Bonde, 2021).

Mortality due to CSS represents about 10% of annual Florida manatee deaths of known cause (based on data from 2012 to 2021; Florida Fish and Wildlife Commission, 2023) but can spike in particularly cold winters (Hardy *et al.,* 2019). Record numbers of cold-related deaths of manatees were recorded following periods of below-average temperatures during each of the 2009–2010 and 2010–2011 winters, which resulted in the declaration of unusual mortality events (UMEs) (NOAA, 2023). During the 2009–2010 winter UME, 252 of 480 manatee carcasses recovered throughout the state of Florida (52.5%) could be directly attributed to CSS. A further 197 deaths were classified as ‘undetermined’ cause; however, it is likely that the majority of these manatees also perished from CSS (Barlas *et al.,* 2011). In addition, 35 live manatees showing signs of CSS were rescued (57% of which were calves) and many were relocated to warm-water refuges or rehabilitation facilities (Barlas *et al.,* 2011). In the 2010–2011 winter, 119 of the 203 manatee deaths recorded between 18 December 2010 and 13 February 2011 (58%) were attributed to CSS (de Wit *et al.,* 2012; Florida Fish and Wildlife Commission, 2023).

Whilst CSS is a significant threat in particularly cold years, watercraft collision poses a persistent year-round threat to Florida manatees (Lightsey *et al.,* 2006; Rommel *et al.,* 2007; Deutsch & Reynolds, 2012). Between 2012 and 2021, watercraft collisions accounted for over 27% of reported manatee deaths for which cause of death was known (Florida Fish and Wildlife Commission, 2023). Sub-lethal injuries are also common, with collision-related scars reported in 96% of adults, 70% of sub-adults and 34% of calves (Bassett *et al.,* 2020). Although management measures including boat-speed restriction zones and no-vessel zones have been implemented to reduce collisions (Calleson & Kipp Frohlich, 2007), watercraft interactions remains an important threat to Florida manatees (Runge *et al.,* 2017).

Considering the many threats that Florida manatees are exposed to, understanding the physiological consequences of exposure to stressors is crucial for monitoring and prediction of population-level effects, as well as for the design of policy and management initiatives that can help mitigate impacts. As global climate change is predicted to change the frequency, duration and intensity of extreme weather events such as heat waves and cold snaps (Nowicki *et al.,* 2019; IPCC, 2023), and considering the threat of warm-water habitat loss (Runge *et al.,* 2017), Florida manatees are likely to remain vulnerable to episodic cold events while simultaneously facing changes in the availability and distribution of warm-water refugia. Indeed, climate models predict that cold-air outbreaks will continue (Vavrus *et al.,* 2006; Kodra *et al.,* 2011), and species that are susceptible to temperature changes, such as the Florida manatee, are the most likely to suffer negative impacts (Tewksbury *et al.,* 2008). In order to design management and policy guidelines that mitigate the potential threats of climate-related impacts on Florida manatee populations into the future, we first need a solid understanding of how manatees respond to extreme weather events, as well as to other major stressors.

A standard approach to assessing how animals respond to social, physical or environmental challenges is through measuring hormones associated with the stress response and broader physiological regulation (Cooke *et al.,* 2013; McCormick & Romero, 2017). Cortisol and corticosterone are the hormones most commonly measured to assess stress responses in mammals. However, in addition to their role in the stress response (Sapolsky *et al.,* 2000), these adrenal hormones are involved in maintaining homeostasis across a wide range of bodily processes, including metabolism (Romero, 2002; Vegiopoulos & Herzig, 2007), immune function (McEwen *et al.,* 1997), arousal and cognition (Jaszczyk & Juszczak, 2021) and certain aspects of development (Liggins, 1994; Crespi *et al.,* 2013). Consequently, disentangling the causes and effects of fluctuations in cortisol and corticosterone can be challenging, and other hormones such as aldosterone and the thyroid hormones triiodothyronine (T3) and thyroxine (T4), among others, are increasingly being assessed together with corticosteroids in an attempt to gain a more fine-grained understanding of the physiological responses of marine mammals to stressors (St. Aubin *et al.,* 1996; Jeanniard du Dot *et al.,* 2009; Ayres *et al.,* 2012; Burgess *et al.,* 2017; Lemos *et al.,* 2020; Miller *et al.,* 2021).

Aldosterone is an adrenal mineralocorticoid involved in osmoregulation that also plays a role in the stress response by regulating electrolyte and water balance and helping to stabilize blood pressure (Kubzansky & Adler, 2010). Its role in the stress response of marine mammals living in hyperosmotic environments is particularly relevant, with studies on cetaceans finding that blood aldosterone increases along with cortisol in response to stressors such as capture (Thomson & Geraci, 1986; St. Aubin & Geraci, 1989; St. Aubin *et al.,* 1996; Ortiz & Worthy, 2000; Schmitt *et al.,* 2010; Hart *et al.,* 2015), loud noise (Romano *et al.,* 2004) and exposure to cold water (Houser *et al.,* 2011). In right whales, faecal aldosterone appears to be positively correlated with faecal corticosteroids and is elevated in reproductively active adults (Burgess *et al.,* 2017). In Florida manatees, plasma aldosterone has been found to vary with sampling location, with manatees from brackish conditions in the Indian River Lagoon showing higher concentrations than those from fresh water in Crystal River, possibly reflecting differences in salinity and freshwater availability between sites (Brammer-Robbins *et al.,* 2023). Little is known about the broader role of aldosterone in the stress responses of manatees, however, and further work is needed to shed light on the types of stressors that elicit aldosterone secretion and whether faecal aldosterone metabolite concentrations can be used as reliable markers of physiological state.

Thyroid hormones also play a central role in regulating metabolic rate and energy substrate utilization, increasing lipid turnover by stimulating both lipogenesis and lipolysis. Although not primary mediators to the stress response, thyroid hormones interact with stress physiology to influence energy mobilization, with elevated thyroid hormone concentrations generally associated with increased energy expenditure and net lipid loss (Oppenheimer *et al.,* 1991). Distinct patterns in thyroid hormone regulation have been described in response to two types of metabolic challenge. When energy consumption outweighs its availability, such as during seasonal declines in food availability, for example, thyroid hormones may be downregulated to reduce metabolic rate and conserve energy (type 1 thyroid allostasis; Chatzitomaris *et al.,* 2017). In contrast, in situations where energy stores must be mobilized to meet increased energetic demands, such as exposure to cold weather, thyroid hormones may be upregulated to facilitate thermogenesis and fuel use (type 2 thyroid allostasis; Chatzitomaris *et al.,* 2017). Thus, triiodothyronine (T3) and thyroxine (T4), the predominant circulating thyroid hormones, can increase in response to food intake and cold exposure (Silva, 2003). T3 is the more biologically active form but has a shorter half-life than T4 (Eales, 1988). T4 circulates in blood at much higher concentrations than T3, with ~80% of T3 produced through the deiodination of T4 in peripheral tissues (Senese *et al.,* 2014); this deiodination has been found to increase in response to lowered temperatures (Eales, 1988; Silva, 2003).

Hormones and their metabolites can be measured in a variety of animal body tissues, and each approach provides information across a different time period. Blood and saliva provide a recent snapshot of the physiological status of an animal, but the collection process itself may induce a stress response (Cook *et al.,* 2000). Faecal hormone metabolite concentrations, in contrast, reflect an integration of hormones over a period of time (that may be related to hormone metabolism and gut passage time) and are thus particularly useful in assessing the impact of stressors over the longer term (Goymann, 2005; Palme *et al.,* 2005). Faecal samples can often be collected using minimally invasive techniques and are now widely employed in wildlife endocrine research (e.g. Sheldon *et al.,* 2012; Burgess *et al.,* 2013; Gesquiere *et al.,* 2018; Hunt *et al.,* 2019; Hunninck *et al.,* 2020; Lemos *et al.,* 2020; Miller *et al.,* 2021).

The major aim of this study was to analyse faecal cortisol, aldosterone, T3 and T4 metabolite concentrations as potential indicators of physiological changes associated with CSS in Florida manatees. The specific objectives were to (i) characterize baseline faecal adrenal and thyroid hormone metabolite concentrations in apparently healthy live Florida manatees and investigate whether concentrations varied with individual characteristics including sex, size class and reproductive state; and (ii) compare individual faecal hormone concentrations and multivariate hormone profiles among manatees of different health status, namely apparently healthy, dead from CSS and dead from acute watercraft collision, while accounting for the potential influence of age class and sex on these comparisons.

We hypothesized that manatees suffering from CSS would likely exhibit signs of increased thermoregulatory effort, indicated by elevated faecal T3 concentrations, in addition to signs of a generalized stress response, including elevated cortisol. Given the limited understanding of aldosterone’s role in manatee stress responses, we predicted that faecal aldosterone concentrations would follow a similar pattern to cortisol, being elevated in stressed manatees relative to healthy individuals, consistent with findings in other marine mammals (St. Aubin *et al.,* 1996; Schmitt *et al.,* 2010; Hart *et al.,* 2015; Burgess *et al.,* 2017). As T4 serves as the primary precursor to T3, we predicted that faecal T4 concentrations would reflect a similar pattern to T3, with cold-stressed manatees showing altered concentrations relative to healthy individuals consistent with increased thermoregulatory demand. In contrast, healthy free-ranging Florida manatees spending time in warm-water refugia in winter would likely have lower thermoregulatory requirements. Manatees that died of acute injuries from watercraft collisions were likely to have been healthy prior to their fatal injuries and would not be expected to survive long enough for chronic physiological responses to be integrated into faecal samples. Thus, faecal hormone metabolite concentrations in this group were expected to be similar to those in apparently healthy manatees. This group therefore provided a post-mortem comparison for evaluating potential effects associated with sample collection from deceased animals.

## Materials and methods

### Sample collection

#### Apparently healthy Florida manatees

Fresh faecal samples were collected from 60 wild, apparently healthy Florida manatees (*Trichechus manatus latirostris*) at Three Sisters Springs, in the Crystal River National Wildlife Refuge (CRNWR), Florida USA, under US Fish and Wildlife Service permit MA791721–4 issued to the US Geological Survey (USGS) Biological Resources Division Sirenia Project and under The University of Queensland Animal Ethics ANRFA 2023/AE000332. These included 14 adult males, 11 sub-adult males, 4 male calves, 26 adult females and 5 sub-adult females. Of the adult females, 9 were classified as pregnant based on vulval and caudal abdominal distension, and 10 were classified as lactating based on the presence of a dependent calf and distended teats.

Sampling was conducted during the boreal winters of 2010 and 2011 (16 February–25 March, 2010, and 19 November–15 February, 2011). Crystal River is a spring-fed freshwater river that serves as an important natural warm-water refuge for Florida manatees during winter months (Valade *et al.,* 2020). Sampling occurred in areas adjacent to, but outside, designated warm-water sanctuary zones. Individual manatees were approached by a permitted researcher, and freshly excreted faeces protruding from the rectum were collected by hand via snorkelling or duck-diving. This allowed direct assignment of each sample to an identified individual. Sex, apparent overall health condition and female reproductive state were determined by visual assessment. Size class was defined as adult (*>*264 cm), sub-adult (235–*2*64 cm) or calf (*<*235 cm), following Bonde *et al.* (2012) (see Table A.1 in Supplementary Materials).

#### Deceased Florida manatees

Faecal material was collected from the distal colon of deceased wild manatees recovered throughout Florida by the Florida Fish and Wildlife Conservation Commission (FWC) and transported to the FWC Marine Mammal Pathobiology Laboratory (St Petersburg, Florida). Sampling occurred during the boreal winters of 2010 and 2011 (19 February–24 March 2010 and 3 November 2010–15 February 2011; see Table A.1 in Supplementary Materials; Fig. 1).

**Figure 1:**
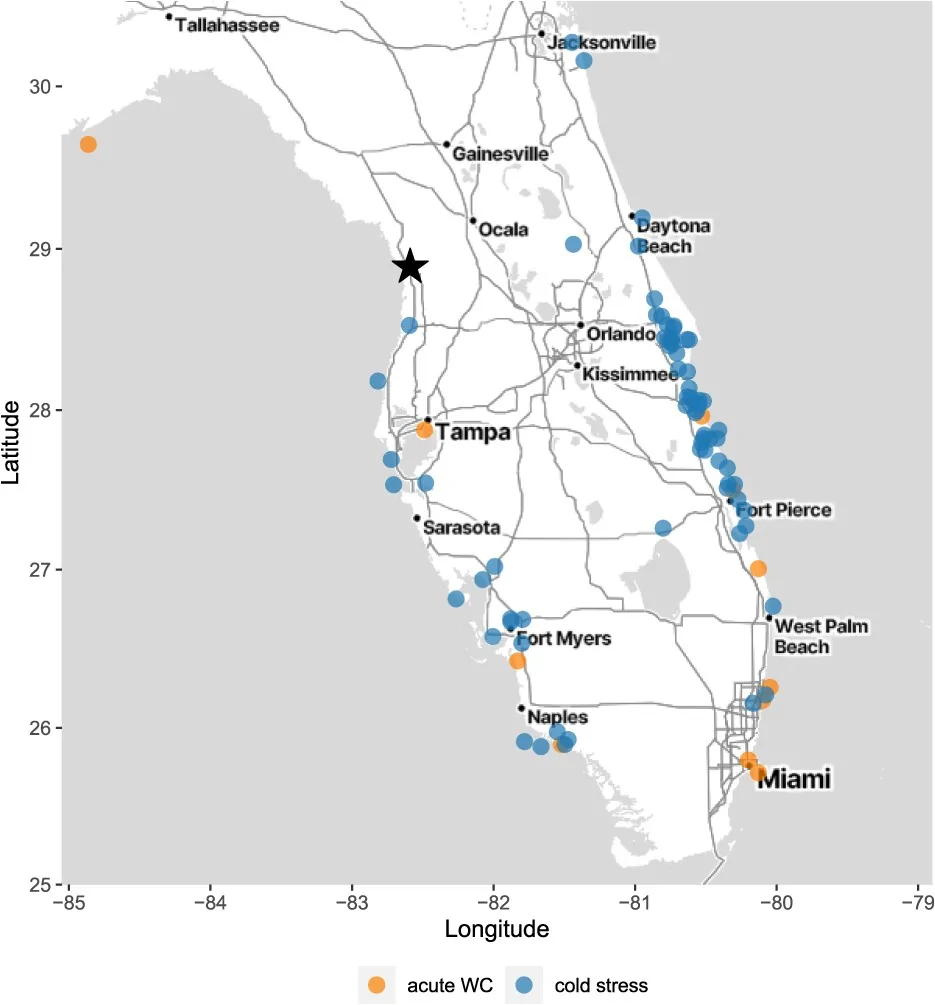
Map of Florida showing the locations of recovered carcasses of Florida manatees that died from cold stress syndrome (CSS) and acute watercraft collision (WC ). The black star marks the Crystal River National Wildlife Refuge, where all samples from live, apparently healthy manatees were collected.

Necropsies were conducted by authorized personnel in accordance with the US Endangered Species Act (50 Code of Federal Regulations (CFR) 17.21(c) and 50 CFR 17.31(b)) and the Marine Mammal Protection Act (50 CFR 18.22). Cause of death was assigned based on pathological findings following standardized necropsy procedures (Bonde *et al.,* 1983). Watercraft-related mortalities were classified as acute or chronic injuries based on the presence or absence of lesion healing (Lightsey *et al.,* 2006). Only individuals attributed to CSS or acute injuries from watercraft collision were included in analyses.

The CSS group comprised 72 manatees, including 6 adult males, 11 sub-adult males, 26 male calves, 3 adult females (including one lactating female), 5 sub-adult females and 21 female calves. The watercraft collision group comprised 11 manatees, including 2 adult males, 3 sub-adult males, 2 male calves, 3 adult females and 1 sub-adult female (see Fig. 4 and Tables A.2 and A.3 in Supplementary Materials for details). Ancillary data included carcass recovery date and location, sex and straight-line total body length (cm) for size class determination. Size class followed the same criteria used for live animals (see above). Carcass condition was categorized as fresh, moderately decomposed or badly decomposed based on external appearance (Bonde *et al.,* 1983; Barlas *et al.,* 2011 report that ‘Carcasses were diagnosed as “Undetermined; Too Decomposed” when stage of decomposition precluded assessment of external lesions and/or presence of significant internal findings’). Most carcasses were moderately decomposed (*n* = 44, 53%), with 14 fresh (16.9%) and 25 badly decomposed (30.1%). To assess potential effects of decomposition on hormone concentrations, values were compared across decomposition categories using a Kruskal–Wallis test. No significant differences were detected for any hormone; therefore, all samples were retained for subsequent analyses.

### Faecal hormone extraction and analysis

Faecal samples were stored at −20**°**C until analysis in 2022. For hormone extraction, samples were oven-dried at 55**°**C overnight and pulverized to produce a homogeneous matrix prior to extraction (Yehle *et al.,* 2025). Approximately, 0.2 ± 0.01 g of dried material was weighed into glass scintillation vials, and 4 ml of 80% methanol (Merck, Germany) was added. Vials were vortexed for 30 s and extracted overnight on a rotating mixer at room temperature. Samples were centrifuged at 1000 × *g* for 10 min and the resulting supernatant was pipetted into a clean vial. Aliquots (500 μl) of extract were evaporated, reconstituted in 500 μl of Arbor Assays® assay buffer and stored at −20**°**C until analysis. Faecal cortisol metabolites (fC) were quantified using Arbor Assays® ISWE Cortisol Mini-Kits (ISWE002) at a 1:4 dilution. Aldosterone metabolites (fA) were quantified using DetectX® Aldosterone Enzyme Immunoassay Kits (K052-H5), also at a 1:4 dilution. Total faecal triiodothyronine metabolites (fT3) were measured using Arbor Assays® DetectX® Triiodothyronine Enzyme Immunoassay Kit (K056-H5), and total faecal thyroxine metabolites (fT4) were measured using Arbor Assays® DetectX® Thyroxine Enzyme Immunoassay Kit (K050-H5), both at a 1:6 dilution. All assays followed manufacturer protocol using a 2-hour incubation format.

### Analytical validation of assays

Assay validation included tests of parallelism and precision. Parallelism was assessed by serially diluting a pool of faecal extracts and statistically comparing the slope of the resulting curve to that of the standard curve (*F*-test *P* > 0.05; Fleetwood *et al.,* 2015). Precision was monitored by using high- (30% binding) and low-concentration (70% binding) control samples on all assay plates. Samples were run in duplicate, and any samples showing intra-assay coefficients of variation exceeding 10% were reanalysed. Inter-assay coefficients of variation were required to be <15%.

**Table 1:** Analytical validation results (parallelism and inter-assay coefficient of variation) for Florida manatee faecal cortisol, aldosterone, total T3 and total T4 metabolite assays

		**Coefficient of variation (%)**	
**Faecal hormone metabolite**	**Parallelism (*F*-test (d** _**f**_ **); *P*-value)**	**Low control (70% binding)**	**High control (30% binding)**
Cortisol	*F*(_1,10_) = 0.56 *P* = 0.47	5.07	13.01
Aldosterone	*F*(_1,10_) = 0.03 *P* = 0.87	12.42	10.42
T3	*F*(_1,10_) = 0.04 *P* = 0.84	7.92	13.84
T4	*F*(_1,10_) = 0.40 *P* = 0.54	9.62	14.72

### Statistical analyses

Descriptive statistics are reported as mean *±* SD. To characterize baseline faecal adrenal and thyroid hormone metabolite concentrations in live, apparently healthy manatees and investigate whether concentrations varied with individual characteristics, differences across sexes were assessed using Mann–Whitney–Wilcoxon tests. Differences across reproductive states (pregnant, lactating and non-reproductive) and size classes (calf, sub-adult and adult) were assessed using Kruskal–Wallis tests with *post hoc* Dunn’s tests and Bonferroni correction.

To characterize the multivariate hormone profiles of each of the three groups and assess whether groups differed in their overall endocrine state, a principal component analysis (PCA) was conducted on the four faecal hormone variables using the *psych* package (Revelle, 2025) in RStudio *(*version 2023.3.0.386; RStudio Team*,* 2023). Hormones were standardized to *z*-scores prior to analysis. No rotation was applied, and principal component scores were extracted for all individuals with complete hormone data. The first two principal components (PC1 and PC2) were retained for downstream analyses. To test whether health groups differed in their multivariate hormone profiles, a one-way multivariate analysis of variance (MANOVA) was conducted on PC1 and PC2 scores with health status as the grouping factor. An additional PCA excluding T4 was also conducted given the known cross-reactivity of the T4 assay with reverse T3 (rT3), which may have inflated T4 values and obscured multivariate structure.

To assess the effects of health status, size class and sex on faecal hormone metabolite concentrations, generalized linear models (GLMs) with a gamma distribution and log link function were fitted separately for each hormone (fC, fA, fT3 and fT4) in RStudio (version 2023.3.0.386; RStudio Team, 2023) using the base stats package. Each model included all individuals with available data for the respective hormone. The gamma distribution was selected to accommodate the right-skewed, strictly positive distribution of hormone concentration data. Each model took the form hormone ~ health status + size class + sex. Initial models included an interaction term, health status × size class; however, model selection using Akaike’s information criterion (AIC) indicated that additive models provided equal or better fit (ΔAIC < 3 for all hormones), and the interaction term did not reach significance for any hormone. Additive models were therefore used for final analyses. Where significant main effects were detected, *post hoc* pairwise comparisons between groups were conducted using estimated marginal means (emmeans package; Lenth, 2023) with Bonferroni correction.

To assess the robustness of findings to two potential confounds—disproportionate representation of calves in the CSS group and potential effects of advanced decomposition on faecal hormone stability—all GLM models were repeated on the same data subsets used for the PCA analyses: (i) excluding badly decomposed carcasses, (ii) excluding calves and (iii) excluding both simultaneously.

## Results

### Analytical validation of assays

All four hormone assays (fC, fA, fT3 and fT4) demonstrated good parallelism with their respective standard curves (see Table 1, Fig. 2), supporting their analytical suitability for measuring immunoreactive hormone metabolites in Florida manatee faecal extracts.

**Figure 2:**
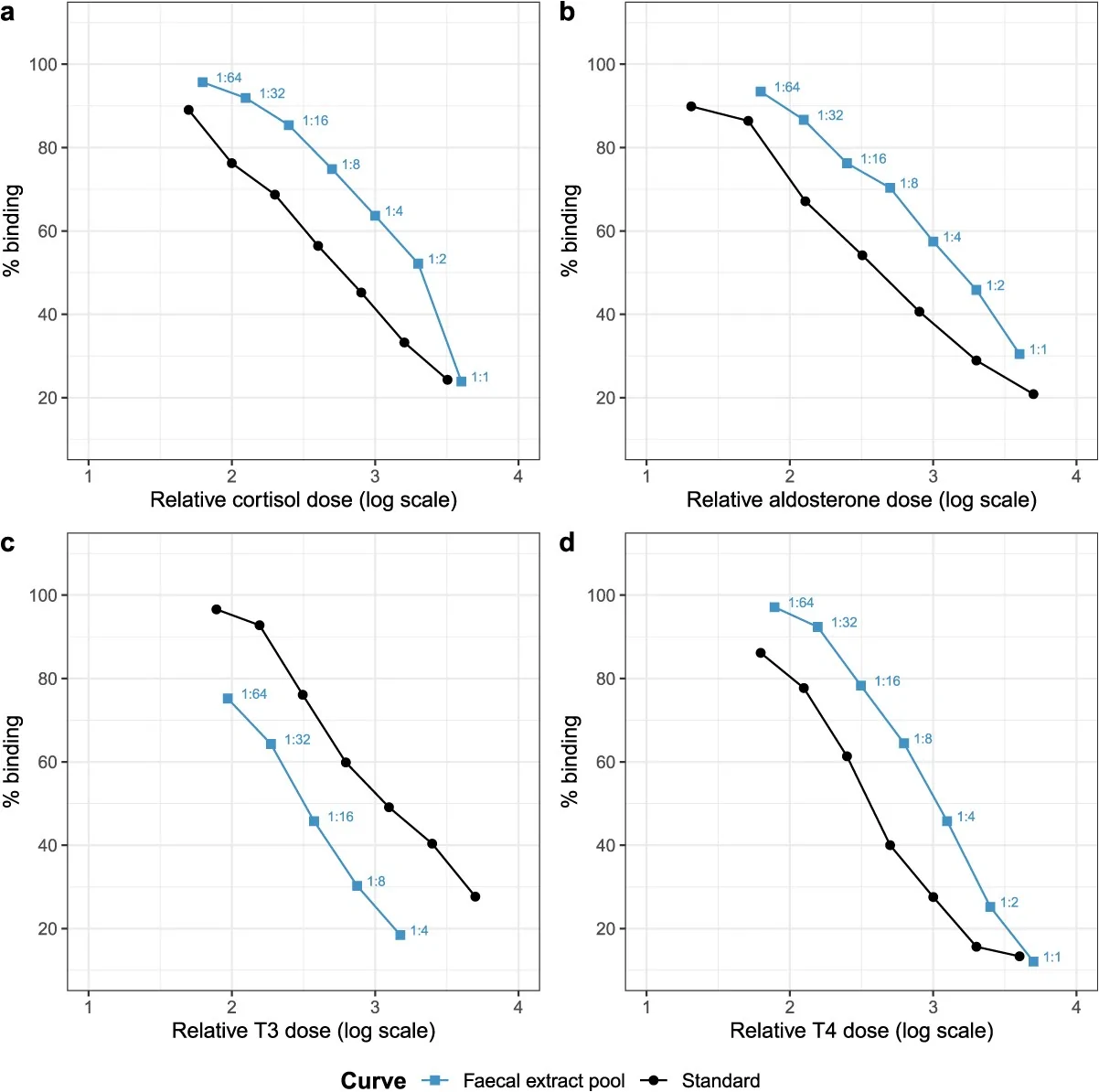
Parallelism graphs showing percentage binding of (a) cortisol, (b) aldosterone, (c) triiodothyronine (T3) and (d) thyroxine (T4) hormone standards against serial dilutions of Florida manatee faecal extracts.

### Winter faecal hormone concentrations in apparently healthy manatees

Descriptive statistics for faecal hormone metabolite concentrations in each group are presented in Table 2. Faecal aldosterone was the only hormone to differ significantly by sex in apparently healthy manatees, with higher concentrations in males than females (Mann–Whitney–Wilcoxon, *W* = 166, *P* = 0.01; males: 18.5 *± 11.7* ng g^−1^ (range 7.6–37.1 ng g^−1^); females: 11.8 *± 7.2* ng g^−1^ (range 4.6–37.2 ng g^−1^)). No statistically significant differences in hormone concentrations were detected among size classes or reproductive states in apparently healthy manatees.

**Table 2:** Descriptive statistics for faecal cortisol, aldosterone, total T3 and total T4 in healthy Florida manatees and manatees that died from CSS and acute watercraft (WC) injuries (ng g^−1^)

**Group**	**Hormone**	***n***	**Range**	**Mean (SD)**
Healthy	Cortisol	47	2.3–19.6	7.2 (3.6)
	Aldosterone	48	4.6–37.2	15.1 (10.2)
	T3	57	31.7–508.9	147.2 (93.9)
	T4	53	5.4–233.9	34.2 (35.1)
Acute WC	Cortisol	11	1.2–40.5	10.2 (11.7)
	Aldosterone	10	4.5–20.9	13.5 (4.9)
	T3	11	33.9–1389.6	266.7 (383.7)
	T4	11	11.5–122.5	38.7 (30.1)
Cold stress	Cortisol	66	1.7–120.4	15.6 (16.3)
	Aldosterone	68	2.6–78.3	23.8 (15.9)
	T3	59	29.5–4086.6	471.5 (630.4)
	T4	70	2.1–310.2	65.5 (67.1)

### Multivariate hormone profiles across health groups

PC1 and PC2 together explained 75.2% of total variance in the full dataset (PC1: 53.4%, PC2: 21.8%), and this structure was stable across sensitivity analyses (PC1 + PC2: 75.2–77.0%). PC1 reflected a general axis of increasing hormone concentrations across all four hormones, whereas PC2 reflected a contrast between adrenal (positive loadings) and thyroid hormones (negative loadings; Table 3).

**Table 3:** PCA loadings for faecal hormone profiles using the full dataset (bold values indicate loadings ≥0.50)

**Variable**	**PC1**	**PC2**	**PC3**	**PC4**
Faecal cortisol	**0.708**	0.387	**−0.560**	0.189
Faecal aldosterone	**0.632**	**0.559**	**0.535**	0.028
Faecal T3	**0.712**	**−0.593**	0.170	0.335
Faecal T4	**0.853**	−0.241	−0.074	−0.458
Eigenvalue	2.135	0.872	0.634	0.359
% variance explained	53.4	21.8	15.9	9.0
Cumulative % variance	53.4	75.2	91.0	100.0

Health status had a significant multivariate effect on PC1 and PC2 scores in the full dataset (MANOVA: Pillai = 0.170, *F*_4,180_ = 4.17, *P* = 0.003; *n* = 93), with CSS manatees showing higher PC1 scores than healthy and acute WC manatees, although considerable overlap was observed between groups in the biplot (Fig. 3). This effect was sensitive to dataset composition. The multivariate effect strengthened when badly decomposed carcasses were excluded (Pillai = 0.224, *F*_4,142_ = 4.49, *P* = 0.002; *n* = 74) but became non-significant after excluding calves (Pillai = 0.134 F_4,96_ = 1.72, *P* = 0.152; *n* = 51) or both calves and badly decomposed carcasses (Pillai = 0.082 *F*_4,84_ = 0.900, *P* = 0.468; *n* = 45).

**Figure 3:**
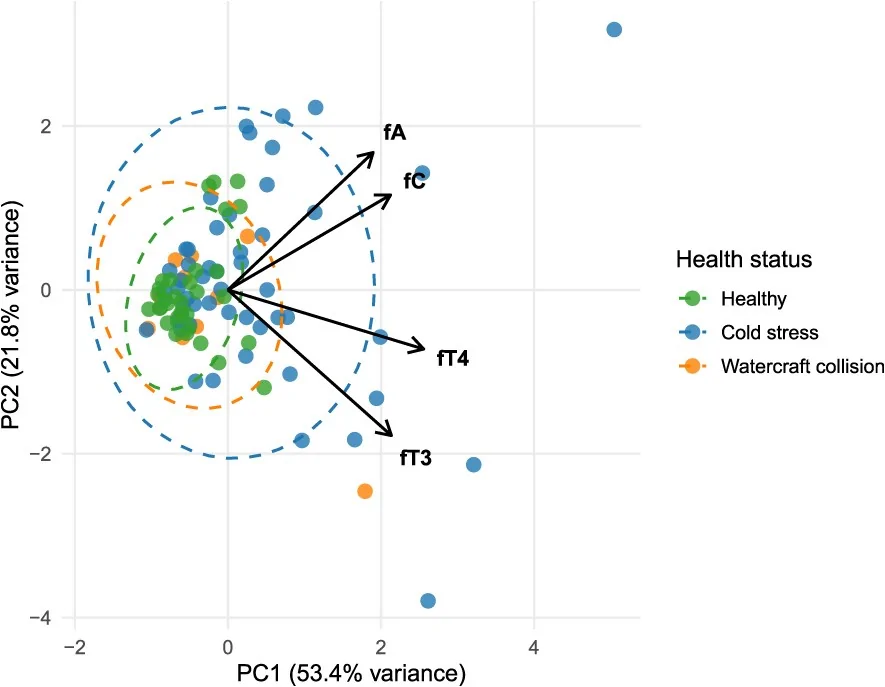
PCA biplot of faecal hormone profiles by health status in manatees. Ellipses represent 95% CIs. Arrows indicate hormone loadings on PC1 and PC2. Note that the small watercraft collision ellipse reflects the limited sample size.

When fT4 was excluded from the PCA in recognition of its known cross-reactivity with rT3, the variance explained by PC1 and PC2 increased to 79.2% (Table 4), and the multivariate effect of health status strengthened (Pillai = 0.202, *F*_4,186_ = 5.22, *P* < 0.001; *n* = 96), although considerable overlap among groups remained (Fig. 4). As in the analysis including fT4, the multivariate effect was stronger when badly decomposed carcasses were excluded (Pillai = 0.268, *F*_4,148_ = 5.72, *P* < 0.001; *n* = 77). In contrast to the analysis including fT4; however, the effect remained significant after excluding calves alone (Pillai = 0.189, *F*_4,100_ = 2.61, *P* = 0.04; *n* = 53), although the effect was weaker and became non-significant only when both calves and badly decomposed carcasses were excluded (Pillai = 0.093, *F*_4,88_ = 1.07, *P* = 0.377; *n* = 47).

**Table 4:** PCA loadings for faecal hormone profiles excluding fT4 and using the full dataset (bold values indicate loadings ≥0.50)

**Variable**	**PC1**	**PC2**	**PC3**
Faecal cortisol	**0.776**	−0.306	**0.552**
Faecal aldosterone	**0.785**	−0.255	**−0.564**
Faecal T3	**0.508**	−0.306	**0.552**
Eigenvalue	1.476	0.900	0.624
% variance explained	49.2	30.0	20.8
Cumulative % variance	49.2	79.2	100.0

**Figure 4:**
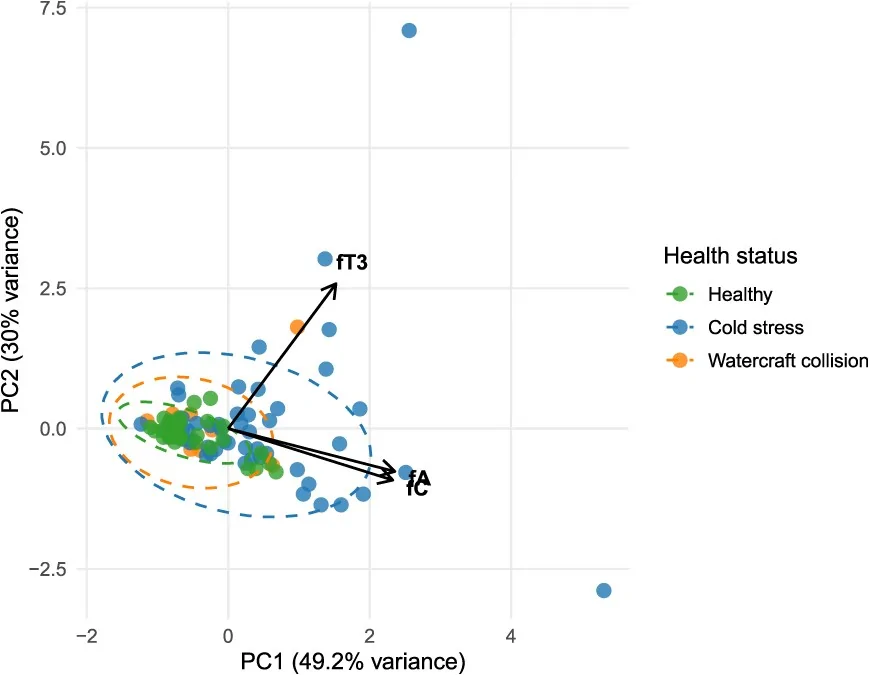
PCA biplot of faecal hormone profiles by health status in manatees, excluding fT4. Ellipses represent 95% CIs. Arrows indicate hormone loadings on PC1 and PC2.

### Effects of health status, size class and sex on faecal hormone concentrations GLMs

#### Faecal cortisol metabolites

No significant main effects of health status or sex were detected for faecal cortisol metabolite concentrations (fC) (health: *χ^2^₂* = 2.55, *P* = 0.279; sex: *χ^2^₁* = 0.025, *P* = 0.874; Table 5). Size class was significant (*χ^2^₂* = 8.44, *P* = 0.015), with calves showing higher fC than adults (ratio = 2.00, *P* = 0.008) and sub-adults (ratio = 1.70, *P* = 0.037). However, this result was not maintained when calves were excluded from the analyses (Table 5).

**Table 5:** Results of GLMs examining effects of health status, size class and sex on faecal hormone metabolite concentrations in Florida manatees

		**Cortisol**		**Aldosterone**		**T3**		**T4**	
**Dataset**	**Predictor**	**χ** ^**2**^	***P***	**χ** ^**2**^	***P***	**χ** ^**2**^	***P***	**χ** ^**2**^	***P***
Full dataset (*n* = 143)	Health status	2.55	0.279	6.25	**0.044**	16.50	**<0.001**	7.58	**0.023**
	Size class	8.44	**0.015**	2.84	0.241	3.01	0.222	3.42	0.181
	Sex	0.025	0.874	3.21	0.073	0.060	0.807	0.133	0.715
Excl. badly decomposed (*n* = 118)	Health status	3.09	0.214	2.08	0.354	14.55	**<0.001**	6.71	**0.035**
	Size class	9.84	**0.007**	1.99	0.370	3.54	0.170	2.76	0.251
	Sex	0.021	0.884	3.74	0.053	0.002	0.962	1.25	0.264
Excl. calves (*n* = 90)	Health status	4.81	0.090	2.78	0.249	19.78	**<0.001**	3.92	0.141
	Size class	2.00	0.158	2.72	0.099	2.87	0.090	2.56	0.110
	Sex	1.83	0.177	2.18	0.140	2.01	0.156	2.80	0.094
Excl. both (*n* = 72)	Health status	1.00	0.607	0.31	0.856	16.00	**<0.001**	2.85	0.240
	Size class	0.173	0.677	1.75	0.186	2.58	0.108	2.20	0.138
	Sex	0.092	0.761	4.05	**0.044**	1.44	0.231	3.04	0.081

Significant *P*-values are indicated in bold. Dataset *n* indicates the total number of individuals represented in each analysis subset; sample sizes for individual hormone models varied according to hormone data availability.

#### Faecal aldosterone metabolites

Health status was a significant predictor of faecal aldosterone metabolite concentrations (fA) (*χ^2^₂* = 6.25, *P* = 0.044), with CSS manatees showing higher model-estimated concentrations than healthy animals (CSS: 22.1 ng g^−1^, 95% confidence interval (CI) 18.3–26.6; healthy: 15.5 ng g^−1^, 95% CI 12.7–19.0). However, pairwise comparisons did not identify significant differences between any specific health group pairs after Bonferroni correction (healthy vs CSS: *P* = 0.074; all other comparisons *P* > 0.12). Sex was marginally non-significant in the primary model (*χ^2^₁* = 3.21, *P* = 0.073), with males tending toward higher fA concentrations than females (males: 18.7 ng g^−1^, 95% CI 15.6–22.3; females: 15.1 ng g^−1^, 95% CI 12.3–18.6), and reached significance when both calves and badly decomposed carcasses were excluded (*χ^2^₁* = 4.05, *P* = 0.044; Table 5). The effect of health status on fA was not robust to sensitivity analyses (badly decomposed carcasses excluded: *χ^2^₂* = 2.08, *P* = 0.354; calves excluded: *χ^2^₂* = 2.78, *P* = 0.249; both excluded: *χ^2^₂* = 0.31, *P* = 0.856; Table 5).

#### Faecal T3 metabolites

Health status was a highly significant predictor of faecal T3 metabolite concentrations (fT3) (*χ^2^₂* = 16.50, *P* < 0.001). Pairwise comparisons revealed significantly higher fT3 in CSS manatees compared to healthy animals (ratio = 3.12, *P* < 0.001), while acute WC manatees did not differ from either healthy or CSS manatees (all *P* > 0.33). Model-estimated mean fT3 concentrations were 445.2 ng g^−1^ (95% CI 323.3–613.1) in CSS manatees, 250.4 ng g^−1^ (95% CI 133.0–471.5) in acute WC manatees and 142.8 ng g^−1^ (95% CI 103.3–197.3) in healthy manatees. No significant effects of size class or sex were detected (size class: *χ^2^₂* = 3.01, *P* = 0.222; sex: *χ^2^₁* = 0.060, *P* = 0.807). The significant health status effect was robust across all sensitivity analyses: excluding badly decomposed carcasses (*χ^2^₂* = 14.55, *P* < 0.001; healthy vs CSS: *P* = 0.0003), excluding calves (*χ^2^₂* = 19.78, *P* < 0.001; healthy vs CSS: *P* < 0.001) and excluding both (*χ^2^₂* = 16.00, *P* < 0.001; healthy vs CSS: *P* = 0.002) (Table 5). Individual concentrations of fT3 (and the other measured hormones) by health status and size class are illustrated in Fig. 5.

**Figure 5:**
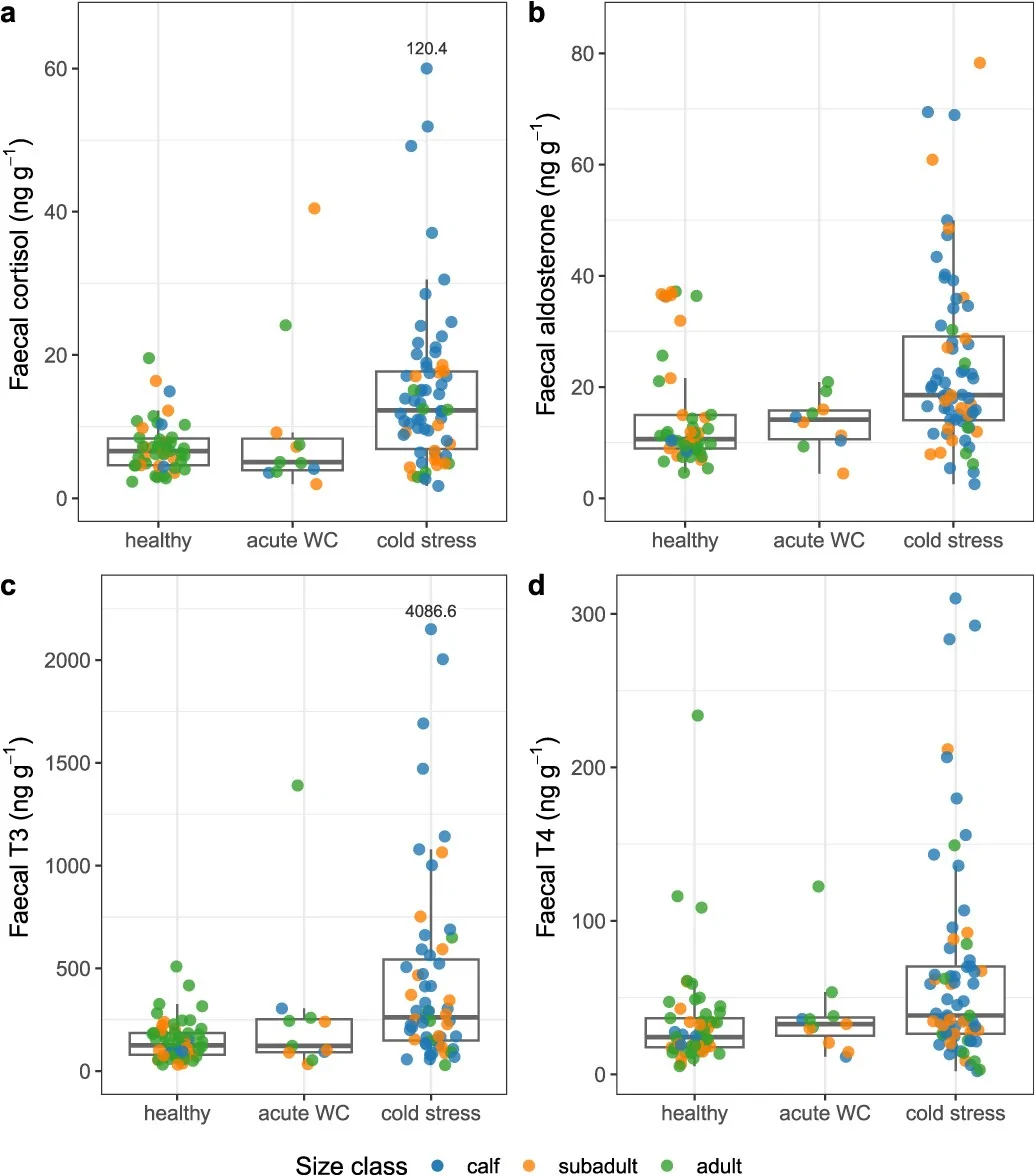
Faecal hormone metabolite concentrations (ng g^−1^) by health status and size class in Florida manatees. Box plots show median and inter-quartile range. For panels **a** and **c**, the *y*-axis is truncated for visual clarity. Values exceeding the axis maximum are indicated by a data point at the axis limit with the true value labelled above.

#### Faecal T4 metabolites

Health status was a significant predictor of faecal T4 metabolite concentrations (*χ^2^₂* = 7.58, *P* = 0.023). Pairwise comparisons revealed significantly higher fT4 in CSS manatees compared to live, healthy animals (ratio = 1.93, *P* = 0.010), whereas acute WC manatees did not differ significantly from either group (all *P* > 0.33). Model-estimated mean fT4 concentrations were 63.1 ng g^−1^ (95% CI 48.4–82.1) in CSS manatees, 37.2 ng g^−1^ (95% CI 20.8–66.5) in acute WC manatees and 32.7 ng g^−1^ (95% CI 24.3–43.9) in live, healthy manatees. No significant effects of size class or sex were detected (size class: *χ^2^₂* = 3.42, *P* = 0.181; sex: *χ^2^₁* = 0.133, *P* = 0.715) (Table 5). The effect of health status was retained when badly decomposed carcasses were excluded (*χ^2^₂* = 6.71, *P* = 0.035; healthy vs CSS: *P* = 0.019), but was not significant after excluding calves (*χ^2^₂* = 3.92, *P* = 0.141) or after both exclusions (*χ^2^₂* = 2.85, *P* = 0.240) (Table 5).

## Discussion

We found that faecal thyroid hormone concentrations were associated with health status in manatees. Faecal T3 showed the strongest and most consistent association with health status, with CSS manatees exhibiting concentrations ~3 times higher than apparently healthy animals across all sensitivity analyses. Faecal T4 also differed significantly between CSS and healthy manatees, though this result was less robust to sensitivity testing. Faecal aldosterone showed a marginal effect of health status in the primary model that was not maintained in sensitivity analyses, while faecal cortisol variation was more strongly associated with size class than health status. These findings suggest that faecal T3 may be a promising candidate endocrine biomarker of CSS in Florida manatees.

### Winter faecal hormone metabolite concentrations in apparently healthy manatees

Apparently healthy Florida manatees in this study were all sampled in Three Sisters Springs in Crystal River, Florida, which is part of the CRNWR that was established in 1983 with the specific goal of protecting manatees (Slone *et al.,* 2023). The Crystal River Springs complex is an established primary warm-water refugia with water temperatures above 22°C even during severe cold weather and is designated as a seasonal no-entry zone, which limits anthropogenic disturbance in winter when manatees are present in high numbers (Valade *et al.,* 2020). Given the role of cortisol in the stress response, the relatively low mean fC concentration of 7.2 *±* 3.6 ng g^−1^ (range 2.3–19.6 ng g^−1^) observed in these manatees may reflect reduced allostatic load, given their access to thermally suitable habitat and limited anthropogenic disturbance. Similarly, mean faecal aldosterone metabolite concentrations (fA) of 15.1 *±* 10.2 ng g^−1^ (range 4.6–37.2 ng g^−1^) in this group may reflect levels in manatees not experiencing stressors sufficient to disrupt cardiovascular or osmotic homeostasis. A recent movement tracking study by Slone *et al.* (2023) demonstrated that manatees within the Crystal River Springs complex and surrounding areas use the landscape strategically, exhibiting habitat-use patterns consistent with efficiently meeting their foraging and thermoregulatory needs while avoiding human interaction. Faecal cortisol and aldosterone metabolite concentrations reported here may represent overwintering levels for Florida manatees in the absence of extreme environmental disturbances, including extreme temperature stressors.

Faecal aldosterone concentrations were significantly higher in males than females among apparently healthy manatees. Sex-related patterns in aldosterone regulation are inconsistent across marine mammal species and are often linked to reproductive or energetic state. In Steller sea lions (*Eumetopias jubatus*), male pups showed higher aldosterone concentrations than female pups, though this difference was not maintained into adulthood (Keogh *et al.,* 2013). In fasting northern elephant seals (*Mirounga angustirostris*), aldosterone increased in fasting adult males during the breeding season, contributing to increased water retention and the regulation of ionic and osmotic homeostasis (Ortiz *et al.,* 2006). In North Atlantic right whales, faecal aldosterone varied with reproductive state rather than sex per se, with reproductively active adults of both sexes showing elevated concentrations relative to non-reproductive individuals, suggesting that aldosterone elevation may serve to increase sodium and water retention, maintaining blood pressure and blood flow in the face of the increased physiological demands of reproduction (Burgess *et al.,* 2017). The biological basis for the higher faecal aldosterone observed in male manatees remains unclear but may relate to differences in body size, osmoregulation or energetic demands and warrants further investigation.

It is not uncommon for manatees overwintering in warm-water refugia to fast for periods of seven to 10 days (Deutsch *et al.,* 2022). While the warm waters of this relatively protected winter-refuge environment allow manatees to conserve energy and reduce the need for drawing upon fat reserves for thermoregulation (Chatzitomaris *et al.,* 2017), this benefit comes at the cost of reduced access to foraging areas, as manatees may commute up to 40 km to feeding grounds from their wintering sites (Deutsch *et al.,* 2022). This trade-off between thermoregulation and nutrition, together with seasonal changes in diet and environmental conditions during overwintering (Merson *et al.,* 2013), is a defining feature of the winter ecology of Florida manatees and is relevant to interpreting the thyroid hormone concentrations observed in apparently healthy animals. As thyroid hormones serve to regulate energy expenditure from metabolic processes such as feeding and thermoregulation, thyroid hormones may be downregulated when Florida manatees are overwintering in warm-water refugia and navigating periods of fasting. This pattern is consistent with type 1 thyroid allostasis, in which thyroid hormone production decreases to conserve energy under conditions of reduced metabolic demand (Chatzitomaris *et al.,* 2017). It is therefore not clear whether the faecal thyroid hormone metabolite concentrations reported here for apparently healthy manatees represent true baseline values or reflect a degree of the downregulation in the context of fasting between winter foraging trips. Changes in circulating thyroid hormone concentrations in response to fasting have been found to differ between species and across life history stages. Ortiz *et al.* (2000) found that Florida manatees under managed care that were subjected to a reduced diet had an increase in circulating T4 after a few days but no concomitant increases in T3, suggesting that deiodinase activity from T4 to the more biologically active T3 was unchanged under mild nutritional restriction. In contrast, a decrease in thyroid hormones was observed in juvenile northern elephant seals that had been fasting for over 3 weeks (Jelincic *et al.,* 2017), but little change was seen across the fasting period in postweaning pups, potentially due to reduced metabolic clearance of T3 and T4 (Ortiz *et al.,* 2003). Similarly, no changes in serum thyroid hormone concentrations were observed in adult male northern elephant seals across their breeding fast, possibly as a result of increased energetic requirements during this time (Crocker *et al.,* 2012). Faecal thyroid hormone concentrations measured at other times of the year, when Florida manatees are able to forage continuously, would help clarify how short-term winter fasts affect thyroid hormone concentrations in this species, and inform interpretation of the values reported here.

### Faecal hormones in cold-stressed manatees

#### Thyroid hormones

Of the hormones examined, faecal T3 showed the strongest and most consistent association with CSS, with significantly elevated concentrations across all analytical subsets. This consistency suggests that faecal T3 is a promising candidate biomarker of CSS in Florida manatees and that altered thyroid hormone activity associated with cold stress is detectable in faecal samples. As with the other hormones examined, individual fT3 values in CSS manatees tended to be higher among calves and sub-adults than adults; this pattern and the potential vulnerability of immature manatees to cold stress are discussed further below.

Elevated fT3 observed in CSS manatees may reflect increased thermoregulatory demands associated with prolonged cold exposure. Thyroid hormones are crucially important for thermoregulation and thermogenesis in homeotherms, and many species including African elephants (*Loxodonta africana*; Szott *et al.,* 2020) and impala (*Aepyceros melampus*; Hunninck *et al.,* 2020) show increases in thyroid hormones as temperatures decline. The cold-stressed manatees in this study were in an advanced state of nutritional compromise, as evidenced by body emaciation and empty digestive tracts characteristic of late-stage CSS (Bossart *et al.,* 2002). While low nutrient intake generally leads to a downregulation of thyroid hormones, the thermoregulatory challenge faced by CSS manatees, compounded by severely depleted fat reserves, may have overridden nutritional suppression of the thyroid axis, driving fT3 upregulation particularly in animals with lower thermal mass and reduced fat thickness. This interpretation is consistent with type 2 thyroid allostasis, in which thyroid hormones are upregulated to mobilize energy reserves in response to cold exposure (Chatzitomaris *et al.,* 2017). However, temperature effects on thyroid hormones appear to be species-specific, with some mammalian species showing no variation in thyroid hormone concentrations with changing temperatures (Dias *et al.,* 2017; Touitou *et al.,* 2021). Additional research is needed to confirm the drivers of elevated fT3 in CSS manatees specifically. One promising approach would be to measure faecal thyroid metabolite concentrations in manatees experiencing starvation without exposure to extreme temperatures, which could help disentangle the respective contribution of starvation and thermoregulatory demand to the thyroid hormone patterns observed here.

It is important to note, however, that an alternative interpretation of elevated fT3 in CSS manatees exists. Chronic physiological stress is known to increase production of rT3, an inactive metabolite of T4 that competes with T3 for receptor binding and can suppress metabolism and reduce energy expenditure during periods of nutritional stress (Halsall & Oddy, 2021). Marine mammals, including cetaceans and pinnipeds, have been found to maintain consistently high levels of rT3 relative to terrestrial mammals, with rT3 concentrations approaching those of T4 in some species (St. Aubin *et al.,* 1996; Ortiz *et al.,* 2003; St. Aubin *et al.,* 2013; Ensminger *et al.,* 2014; Atkinson *et al.,* 2015; Champagne *et al.,* 2015). However, the T3 assay used in this study shows negligible cross-reactivity with rT3 (<0.1%), meaning that the elevated fT3 values reported here are unlikely to be explained by rT3 interference. This supports the interpretation that the observed fT3 elevation reflects T3 immunoreactivity associated with CSS, although the biological identity of the faecal metabolites contributing to this signal cannot be established from the immunoassay alone. Future work using more specific analytical techniques such as liquid chromatography–tandem mass spectrometry (LC–MS/MS) to directly quantify T3, T4 and rT3 metabolites would enable these competing mechanisms to be more fully evaluated.

Faecal T4 concentrations were significantly higher in CSS manatees than in healthy animals in the primary model, though this effect was not maintained when calves were excluded, suggesting it may be partially driven by calf values. Given the substantial cross-reactivity of the T4 assay with rT3, the biological interpretation of these findings remains uncertain, and the fT4 values reported here should be interpreted with considerable caution, as discussed further below.

#### Adrenal hormones

No significant effect of health status on faecal cortisol metabolite concentrations was detected in any model, with size class emerging as the primary predictor, driven by higher concentrations in calves. The lack of a detectable health status effect on faecal cortisol may reflect the chronic, rather than acute, nature of CSS. Prolonged HPA activation can lead to habituation or dysregulation of the cortisol response, potentially reducing the detectability of cortisol elevation in animals in advanced stages of chronic disease (Romero & Wingfield, 2015). Changes in cortisol associated with developmental stage and life-history transitions, including weaning, independence from maternal care and the ongoing maturation of the HPA axis, are well documented in marine mammals (Ortiz *et al.,* 2001; Atkinson *et al.,* 2011; Jelincic *et al.,* 2017). Most Florida manatee calves remain with their mothers for 1 to 3 years (O’Shea & Hartley, 1995; Rathbun *et al.,* 1995; Koelsch, 2001), and the sample of cold-stressed manatees included both dependent calves and recently independent or orphaned calves, whereas apparently healthy calves were all observed in close association with an adult female. Developmental and life-history factors are therefore likely to have contributed to cortisol variation in younger animals across health groups, potentially obscuring any endocrine signal associated specifically with CSS. These results suggest that faecal cortisol alone may not reliably differentiate cold-stressed from apparently healthy manatees across the full age range of the population, and that age class should be carefully considered when interpreting faecal cortisol data in Florida manatees.

Although faecal aldosterone showed a significant overall health status effect in the primary model, this pattern was not maintained in pairwise comparisons after Bonferroni correction, nor in any sensitivity analysis, suggesting that this result should be interpreted with caution. Aldosterone is primarily involved in maintaining cardiovascular homeostasis through water and electrolyte balance and is known to increase in response to a range of stressors in marine mammals, including exposure to cold water (Houser *et al.,* 2011). In bottlenose dolphins exposed to experimentally decreasing water temperatures, circulating aldosterone began increasing even before temperatures reached the animals’ lower critical temperature, with the authors suggesting that peripheral vasoconstriction through activation of the renin–angiotensin–aldosterone system (RAAS) may be particularly important for thermoregulation when blubber stores are insufficient to mitigate heat loss (Houser *et al.,* 2011). Given the manatee’s poor insulation and low tolerance for sub-optimal water temperatures, aldosterone-mediated RAAS activation might be expected in CSS animals, and the absence of a significant health status effect on fA in this study may reflect the chronic nature of CSS, limitations in sample size within specific age and sex categories or the possibility that aldosterone responses to cold stress in manatees are not reliably captured in faecal samples over the timescales represented here. It is also noteworthy that aldosterone promotes cardiac inflammation, fibrosis and hypertrophy (Kubzansky & Adler, 2010; Buffolo *et al.,* 2022), and myocardial degeneration has been observed in cold-stressed manatees (Bossart *et al.,* 2002). Whether increased aldosterone secretion contributes to cardiac disease in CSS manatees represents an interesting avenue for future research.

### The disproportionate vulnerability of immature manatees to cold stress

The disproportionate representation of calves and sub-adults in the CSS mortality cohort, together with the significant size class effect on faecal cortisol (driven by higher concentrations in calves), further highlights the physiological vulnerability of immature Florida manatees to cold stress. Consistent with this, individual hormone concentrations within the CSS group tended to be higher among calves and sub-adults than adults across all four hormones examined. However, the health status × size class interaction was not statistically significant for any hormone, and this pattern should therefore be interpreted as a qualitative trend rather than a demonstrated statistical effect. Calves comprised the largest proportion of the CSS group in this study, consistent with prior reports of elevated calf mortality during cold weather events in Florida (Barlas *et al.,* 2011). Several interacting factors are likely to contribute to this vulnerability. First, immature manatees have lower thermal mass than adults due to their smaller body size, resulting in a higher surface area to volume ratio and therefore greater relative heat loss in cold water (Schmidt-Nielsen, 1984). Second, manatee calves depend on nutrient-rich maternal milk for 1 to 3 years (O’Shea & Hartley, 1995; Rathbun *et al.,* 1995; Koelsch, 2001), and those separated from their mothers before nutritional independence is achieved are likely to have reduced energy reserves available for thermogenesis if faced with a particularly harsh winter. Weisbrod *et al.* (2021) found that CSS was the leading cause of mortality in large calves specifically, attributing this in part to their relative inexperience in independently navigating warm-water refugia and foraging areas during winter, while smaller calves can still rely on their mothers for guidance and nutrition. Third, the immune system of young animals is still developing, rendering calves more susceptible to the secondary infectious diseases that characterize late-stage CSS, including dermatitis, enterocolitis and bronchopneumonia (Bossart *et al.,* 2002). Bossart *et al.* (2002) observed moderate to severe lymphoid depletion in 83% of necropsied cold-stressed Florida manatees, and Walsh *et al.* (2005) found that *in vitro* proliferation of lymphocytes from rescued CSS manatees was impaired compared to healthy free-ranging animals, providing further evidence of the role of immune suppression in CSS pathology. Together, these thermal, nutritional and immunological factors are likely to increase the vulnerability of calves and sub-adults to CSS and may help explain the pattern of higher hormone concentrations observed in immature CSS manatees in this study.

### Faecal hormones in manatees that died from acute watercraft injuries

Manatees that died of acute watercraft collision injuries did not show significantly different faecal hormone concentrations from live, apparently healthy manatees for any of the four hormones measured, as anticipated given that these animals had no documented signs of chronic illness. However, they also did not differ significantly from CSS manatees for fT3 or fT4. The point estimate for fT3 in acute WC manatees fell numerically between the healthy and CSS groups, with a CI wide enough to overlap both, suggesting the lack of significant pairwise differences may partly reflect limited statistical power (*n* = 11) rather than a true intermediate response. An acute perimortem stress response would not be expected to fully account for this pattern, as faecal hormone metabolites reflect systemic hormone exposure over the hours to days preceding defecation and necropsy findings did not indicate a slow death in this group. The inclusion of the acute WC group nevertheless provides an important post-mortem comparison for distinguishing physiological differences associated with cause of death from potential effects of carcass sampling. However, the small sample size and lack of clear separation from either the live, apparently healthy or CSS groups limit the conclusions that can be drawn from this comparison in the present study. Future studies with larger samples of acutely injured animals would help determine whether the apparent intermediate pattern reflects a genuine physiological signal or limited statistical power.

### Methodological considerations

The relative concentrations of total fT3 and total fT4 found in this study deserve particular comment. In all Florida manatees in this study, mean total fT3 was at least four times higher than total fT4. This was unexpected because circulating concentrations of T3, the more active form of thyroid hormone, are usually lower than those of T4 (Hulbert, 2007). Although some diseases can upset the balance between these two hormones (Chopra, 1983), the higher concentrations of total fT3 compared to total fT4 in samples from apparently healthy manatees suggests that illness was not a factor in these results.

A key technical limitation of the T4 assay used in this study is its strong cross-reactivity (89%) with rT3, a by-product of T4 conversion to T3 that binds with receptors without initiating a signalling cascade (Halsall & Oddy, 2021). This cross-reactivity means that the fT4 values reported here likely reflect an unknown combination of true T4 and rT3, which substantially complicates their interpretation. Marine mammals have been found to maintain consistently elevated rT3 relative to terrestrial mammals, with rT3 concentrations approaching those of T4 in some species (St. Aubin *et al.,* 1996; Ortiz *et al.,* 2003; St. Aubin *et al.,* 2013; Ensminger *et al.,* 2014; Atkinson *et al.,* 2015; Champagne *et al.,* 2015), which would make this cross-reactivity particularly consequential in the present study. The stronger multivariate group separation observed when fT4 was excluded from the PCA suggests that inclusion of fT4 may have obscured some of the multivariate structure, although the extent to which this reflects assay cross-reactivity rather than biological variation cannot be determined. Future application of LC–MS/MS approaches capable of separately quantifying T3, T4 and rT3 metabolites will be critical for resolving thyroid hormone dynamics in Florida manatees.

The multivariate separation among the three health groups was similarly sensitive to both age structure and carcass decomposition. In the full hormone panel, the MANOVA effect strengthened when badly decomposed carcasses were excluded but became non-significant when calves were removed. When fT4 was excluded from the PCA, the effect remained significant after excluding calves alone, but became non-significant when both calves and badly decomposed carcasses were excluded. This suggests that fT4 contributed to the age-related component of the multivariate signal, but also shows that the overall separation among health groups was sensitive to the composition of the dataset. Because calves were disproportionately represented among CSS mortalities, the effects of size class and health status cannot be fully disentangled, and the multivariate separation cannot therefore be attributed to CSS independently of age.

It is also worth noting that apparently healthy manatees were sampled from a single winter-refuge location (Three Sisters Springs, Crystal River), while CSS and acute watercraft collision carcasses were recovered from across Florida. Location-specific factors such as salinity, food availability and anthropogenic disturbance have been shown to influence circulating hormone concentrations in healthy manatees (Brammer-Robbins *et al.,* 2023) and may therefore have contributed to differences in faecal hormone concentrations between groups independently of health status. Future studies sampling apparently healthy manatees from multiple locations would help clarify the influence of location on faecal hormone profiles.

Faecal samples in this study were stored at −20**°**C for ~11–12 years between collection (2010–2011) and hormone analysis (2022). Although samples were kept frozen throughout, the possibility of gradual metabolite degradation over this extended period cannot be excluded (Millspaugh & Washburn, 2004; Behringer *et al.,* 2018), particularly given that long-term faecal steroid stability has not been established for this species. Quantitatively estimating the extent of any such degradation is difficult because directly comparable published values are lacking. Importantly, all samples in this study were subject to comparable storage duration and were extracted and assayed together under an identical protocol, so any systematic degradation would be expected to affect groups broadly rather than selectively bias the between-group comparisons that form the basis of our conclusions.

### Conclusions and future directions

This study demonstrates that faecal hormone metabolite concentrations vary with health status, sex and size class in Florida manatees. Among the hormones examined, faecal T3 showed the strongest and most consistent association with CSS, with elevated concentrations in CSS manatees maintained across multiple analytical approaches and data subsets. Multivariate hormone profiles also differed among health groups, although these patterns were sensitive to age structure and carcass decomposition. These findings suggest that faecal T3 monitoring may represent a promising and minimally invasive tool for assessing physiological changes associated with CSS in Florida manatees, particularly given the apparent vulnerability of immature manatees to CSS.

This work has also provided winter reference values of faecal adrenal and thyroid hormones for apparently healthy Florida manatees. Future work should assess how the faecal corticosteroid and thyroid hormone concentrations of live animals vary throughout the rest of the year and in relation to the reproductive hormones, progesterone and testosterone, in order to broaden our understanding of how adrenal and thyroid activation varies based on season, sex, size class and reproductive state. The development of non-invasive monitoring tools based on faecal hormone metabolites could enable these biomarkers to be used for the assessment of individual and population health in Florida manatees, with the potential for early detection of cold stress before severe disease or mass mortality occurs. However, this will require evaluation of hormone concentrations in animals at earlier stages of CSS to determine how endocrine markers evolve throughout the progression of disease, as well as the adoption of more specific analytical methods such as LC–MS/MS to resolve the contributions of individual thyroid hormone metabolites to the faecal signal. Given the projected increase in frequency and intensity of cold weather events in some regions under climate change scenarios (Vavrus *et al.,* 2006; Kodra *et al.,* 2011) and the threat of loss of warm-water refugia associated with the planned closure of coastal power plants (Valade *et al.,* 2020; Reep & Bonde, 2021), understanding the physiological responses of Florida manatees to cold stress and identifying the most sensitive endocrine indicators of that stress remains a priority for the conservation and management of this vulnerable species.

## Supplementary Material

Web_Material_coag083

## Data Availability

All data generated or analysed during this study are included within this published article and its associated Supplementary Materials.
