# Supplementary material for: Faecal adrenal and thyroid hormones as markers of cold stress in Florida manatees (Trichechus manatus latirostris)
Source: Conserv Physiol. 2026 Nov 12;14(1):coag083. doi: 10.1093/conphys/coag083 (PMC13759191; doi:10.1093/conphys/coag083)
Supplement: Supplementary file 1 [file web_material_coag083.docx]

## **Supplementary materials**

**Table A.1.** Descriptions and faecal cortisol (fC), aldosterone (fA), T3 (fT3) and T4 (fT4) metabolite concentrations (ng g^–1^) of apparently healthy wild Florida manatees in sample. Missing hormone data was due to insufficient faecal sample sizes.

| **Manatee ID** | **Collection date** | **Sex** | **Size class** | **Pregnant** | **Lactating** | **County** | **Waterway** | **fC**  (ng g^–1^) | **fA**  (ng g^–1^) | **fT3**  (ng g^–1^) | **fT4**  (ng g^–1^) |
| --- | --- | --- | --- | --- | --- | --- | --- | --- | --- | --- | --- |
|  |  |  |  |  |  |  |  |  |  |  |  |
| *Healthy* |  |  |  |  |  |  |  |  |  |  |  |
|  |  |  |  |  |  |  |  |  |  |  |  |
| MF0017 | 20/3/2010 | female | adult | not pregnant | not lactating | Citrus | Three Sisters Springs | 7.14 | 37.18 | 97.95 | 14.08 |
| MF0018 | 20/3/2010 | male | adult | not pregnant | not lactating | Citrus | Three Sisters Springs |  | 36.38 | 183.66 | 17.60 |
| MF0019 | 20/3/2010 | male | subadult | not pregnant | not lactating | Citrus | Three Sisters Springs | 5.67 | 36.24 | 68.24 | 17.18 |
| MF0020 | 20/3/2010 | male | subadult | not pregnant | not lactating | Citrus | Three Sisters Springs |  | 37.07 | 54.08 | 10.47 |
| MF0021 | 20/3/2010 | female | adult | not pregnant | lactating | Citrus | Three Sisters Springs |  |  | 141.34 | 20.77 |
| MF0022 | 20/3/2010 | male | calf | not pregnant | not lactating | Citrus | Three Sisters Springs | 14.92 | 36.32 | 153.71 | 27.60 |
| MF0023 | 20/3/2010 | male | subadult | not pregnant | not lactating | Citrus | Three Sisters Springs | 7.17 | 36.70 | 239.52 | 17.52 |
| MF0024 | 20/3/2010 | male | subadult | not pregnant | not lactating | Citrus | Three Sisters Springs |  | 36.54 | 102.50 | 6.76 |
| MF0025 | 20/3/2010 | female | adult | not pregnant | lactating | Citrus | Three Sisters Springs | 19.56 | 10.22 | 135.35 | 22.82 |
| MF0047 | 22/3/2010 | male | subadult | not pregnant | not lactating | Citrus | Three Sisters Springs |  | 14.52 | 148.01 | 33.73 |
| MF0048 | 22/3/2010 | male | adult | not pregnant | not lactating | Citrus | Three Sisters Springs | 4.57 | 12.78 |  | 23.81 |
| MF0049 | 23/3/2010 | male | subadult | not pregnant | not lactating | Citrus | Three Sisters Springs | 16.38 | 31.92 | 203.26 | 34.13 |
| MF0050 | 23/3/2010 | male | subadult | not pregnant | not lactating | Citrus | Three Sisters Springs | 3.55 | 14.96 | 79.69 | 18.84 |
| MF0052 | 24/3/2010 | male | adult | not pregnant | not lactating | Citrus | Three Sisters Springs | 4.23 | 10.82 | 50.23 | 14.93 |
| MF0053 | 24/3/2010 | female | subadult | not pregnant | not lactating | Citrus | Three Sisters Springs | 7.38 | 10.23 | 36.98 | 14.86 |
| MF0054 | 24/3/2010 | male | subadult | not pregnant | not lactating | Citrus | Three Sisters Springs | 7.87 | 7.64 | 67.06 | 60.40 |
| MF0056 | 23/3/2010 | male | adult | not pregnant | not lactating | Citrus | Three Sisters Springs |  | 8.30 | 71.70 |  |
| MF0057 | 23/3/2010 | female | subadult | not pregnant | not lactating | Citrus | Three Sisters Springs | 5.59 | 6.91 | 84.79 | 18.29 |
| MF0058 | 24/3/2010 | female | adult | not pregnant | lactating | Citrus | Three Sisters Springs | 11.46 | 7.45 | 108.54 | 34.37 |
| MF0060 | 24/3/2010 | male | calf | not pregnant | not lactating | Citrus | Three Sisters Springs |  | 10.45 | 88.48 | 25.91 |
| 11-007-F2 | 7/1/2011 | male | adult | not pregnant | not lactating | Citrus | Three Sisters Springs | 5.93 | 9.40 | 119.90 | 18.59 |
| 11-007-F6 | 7/1/2011 | male | adult | not pregnant | not lactating | Citrus | Three Sisters Springs |  | 11.79 | 149.16 | 28.57 |
| 11-007-F8 | 7/1/2011 | female | adult | not pregnant | lactating | Citrus | Three Sisters Springs | 8.21 | 15.01 | 166.66 | 40.44 |
| 11-029-F3 | 29/1/2011 | male | subadult | not pregnant | not lactating | Citrus | Three Sisters Springs | 12.23 | 11.92 | 113.76 | 32.41 |
| 11-032-F1 | 1/2/2011 | male | calf | not pregnant | not lactating | Citrus | Three Sisters Springs | 4.39 | 10.33 | 95.64 | 19.33 |
| 10-322-F1 | 19/11/2010 | male | adult | not pregnant | not lactating | Citrus | Three Sisters Springs | 10.56 | 11.90 | 508.89 | 116.07 |
| 10-322-F2 | 19/11/2010 | female | adult | not pregnant | not lactating | Citrus | Three Sisters Springs | 7.27 | 9.78 | 81.85 |  |
| 10-336-F1 | 2/12/2010 | female | subadult | not pregnant | lactating | Citrus | Three Sisters Springs | 4.61 | 11.77 | 197.54 | 29.99 |
| 10-336-F2 | 2/12/2010 | male | subadult | not pregnant | not lactating | Citrus | Three Sisters Springs | 6.25 | 9.94 |  | 18.92 |
| 10-336-F3 | 2/12/2010 | male | adult | not pregnant | not lactating | Citrus | Three Sisters Springs | 6.14 | 9.03 | 186.21 | 48.83 |
| 10-336-F4 | 2/12/2010 | male | subadult | not pregnant | not lactating | Citrus | Three Sisters Springs |  |  | 76.30 |  |
| 10-336-F5 | 2/12/2010 | female | adult | not pregnant | lactating | Citrus | Three Sisters Springs | 8.49 | 7.42 | 247.38 | 59.05 |
| 10-336-F6 | 2/12/2010 | female | subadult | not pregnant | not lactating | Citrus | Three Sisters Springs | 9.80 | 21.58 | 221.98 | 42.81 |
| 10-336-F7 | 2/12/2010 | female | subadult | not pregnant | not lactating | Citrus | Three Sisters Springs | 4.49 | 8.98 | 31.77 | 19.80 |
| 10-341-F1 | 7/12/2010 | female | adult | pregnant | not lactating | Citrus | Three Sisters Springs | 4.59 | 11.24 | 156.60 | 36.64 |
| 10-355-F1 | 21/12/2010 | female | adult | not pregnant | not lactating | Citrus | Three Sisters Springs | 7.27 | 12.49 |  | 33.81 |
| 10-355-F2 | 21/12/2010 | female | adult | not pregnant | not lactating | Citrus | Three Sisters Springs |  | 8.91 | 185.62 | 34.49 |
| 10-363-F2 | 29/12/2010 | male | adult | not pregnant | not lactating | Citrus | Three Sisters Springs | 6.13 |  | 246.15 | 233.85 |
| 10-363-F3 | 29/12/2010 | female | adult | not pregnant | lactating | Citrus | Three Sisters Springs | 6.30 | 7.42 | 178.71 | 49.97 |
| 10-363-F4 | 29/12/2010 | male | calf | not pregnant | not lactating | Citrus | Three Sisters Springs | 10.30 | 8.41 | 103.81 | 24.87 |
| CCR-10-11 | 7/12/2010 | female | adult | pregnant | not lactating | Citrus | Three Sisters Springs |  | 25.64 | 101.49 | 14.23 |
| 10-341-F2 | 7/12/2010 | female | adult | not pregnant | lactating | Citrus | Three Sisters Springs | 4.85 |  | 161.01 | 44.37 |
| 10-341-F3 | 7/12/2010 | female | adult | pregnant | not lactating | Citrus | Three Sisters Springs | 10.26 | 14.33 | 315.61 | 108.66 |
| 10-355-F3 | 21/12/2010 | female | adult | not pregnant | not lactating | Citrus | Three Sisters Springs | 7.05 |  | 57.50 | 5.36 |
| 11-007-F1 | 7/1/2011 | female | adult | not pregnant | lactating | Citrus | Three Sisters Springs | 3.11 | 9.75 | 124.89 | 13.41 |
| 11-007-F3 | 7/1/2011 | female | adult | not pregnant | lactating | Citrus | Three Sisters Springs | 7.20 |  | 31.70 |  |
| 11-007-F4 | 7/1/2011 | female | adult | not pregnant | not lactating | Citrus | Three Sisters Springs | 2.94 | 5.37 | 64.00 |  |
| 11-007-F5 | 7/1/2011 | female | adult | pregnant | not lactating | Citrus | Three Sisters Springs | 5.15 |  | 103.49 | 27.72 |
| 11-007-F7 | 7/1/2011 | female | adult | pregnant | not lactating | Citrus | Three Sisters Springs | 5.71 | 4.59 | 55.77 | 10.29 |
| 11-007-F9 | 7/1/2011 | male | adult | not pregnant | not lactating | Citrus | Three Sisters Springs | 7.77 | 10.31 | 416.77 | 60.98 |
| 11-007-F10 | 7/1/2011 | female | adult | pregnant | not lactating | Citrus | Three Sisters Springs | 6.96 | 9.22 | 205.81 | 22.79 |
| 11-029-F1 | 29/1/2011 | male | adult | not pregnant | not lactating | Citrus | Three Sisters Springs | 8.45 | 21.04 | 326.51 | 47.23 |
| 11-029-F2 | 29/1/2011 | female | adult | pregnant | not lactating | Citrus | Three Sisters Springs |  |  | 58.95 | 14.02 |
| 11-029-F4 | 29/1/2011 | female | adult | not pregnant | not lactating | Citrus | Three Sisters Springs |  | 11.38 | 227.53 | 24.26 |
| 11-040-F1 | 9/2/2011 | female | adult | pregnant | not lactating | Citrus | Three Sisters Springs | 10.77 |  | 282.33 | 18.63 |
| 11-040-F2 | 9/2/2011 | female | adult | pregnant | not lactating | Citrus | Three Sisters Springs | 6.59 | 6.61 | 184.18 | 16.80 |
| 11-040-F3 | 9/2/2011 | female | adult | not pregnant | not lactating | Citrus | Three Sisters Springs | 2.31 | 8.67 | 181.03 | 39.29 |
| 11-046-F1 | 15/2/2011 | male | adult | not pregnant | not lactating | Citrus | Three Sisters Springs | 3.11 |  | 75.96 | 22.17 |
| 11-046-F2 | 15/2/2011 | male | adult | not pregnant | not lactating | Citrus | Three Sisters Springs | 2.78 |  | 89.67 |  |
| 11-046-F3 | 15/2/2011 | male | adult | not pregnant | not lactating | Citrus | Three Sisters Springs | 4.02 |  | 143.83 |  |
|  |  |  |  |  |  |  |  |  |  |  |  |

**Table A.2.** Descriptions and faecal cortisol (fC), aldosterone (fA), T3 (fT3) and T4 (fT4) metabolite concentrations (ng g^–1^) of wild Florida manatees that died of cold stress syndrome. Missing hormone data was due to insufficient faecal sample sizes.

| **Manatee ID** | **Collection date** | **Sex** | **Size class** | **Pregnant** | **Lactating** | **County** | **Waterway** | **fC**  (ng g^–1^) | **fA**  (ng g^–1^) | **fT3**  (ng g^–1^) | **fT4**  (ng g^–1^) |
| --- | --- | --- | --- | --- | --- | --- | --- | --- | --- | --- | --- |
|  |  |  |  |  |  |  |  |  |  |  |  |
| *Cold-stress* |  |  |  |  |  |  |  |  |  |  |  |
|  |  |  |  |  |  |  |  |  |  |  |  |
| LPZ102880 | 2/1/2011 | female | calf | not pregnant | not lactating | Charlotte | Peace River | 2.68 | 2.55 | 57.74 | 21.44 |
| LPZ102879 | 27/12/2010 | male | adult | not pregnant | not lactating | Hernando | Jenkins Creek | 2.96 |  |  | 13.91 |
| MEC10101 | 15/3/2010 | male | subadult | not pregnant | not lactating | Brevard | Indian River | 17.55 | 48.50 | 593.21 | 58.86 |
| MEC10104 | 3/3/2010 | female | calf | not pregnant | not lactating | Brevard | Sykes Creek | 6.39 | 11.56 | 168.97 | 31.57 |
| MEC10105 | 3/3/2010 | male | subadult | not pregnant | not lactating | Indian River | Sebastian River |  | 78.29 | 1064.22 | 67.40 |
| MEC10106 | 4/3/2010 | female | subadult | not pregnant | not lactating | Brevard | Indian River | 7.60 | 11.96 | 87.69 | 28.87 |
| MEC10108 | 4/3/2010 | male | calf | not pregnant | not lactating | Indian River | Atlantic Ocean | 15.85 | 27.70 | 661.67 | 70.44 |
| MEC10110 | 7/3/2010 | female | calf | not pregnant | not lactating | Brevard | Indian River | 17.08 | 11.60 | 126.17 | 39.59 |
| MEC10113 | 8/3/2010 | male | calf | not pregnant | not lactating | Brevard | Indian River | 120.42 | 49.98 | 506.31 | 283.54 |
| MEC10117 | 9/3/2010 | female | calf | not pregnant | not lactating | Brevard | Indian River | 13.24 | 21.55 | 412.35 | 292.46 |
| MEC10118 | 10/3/2010 | male | calf | not pregnant | not lactating | Brevard | Indian River | 21.69 | 26.89 | 1471.19 | 63.73 |
| MEC10121 | 12/3/2010 | male | subadult | not pregnant | not lactating | Volusia | Mosquito Lagoon | 4.30 | 16.84 | 251.50 | 30.23 |
| MEC10122 | 12/3/2010 | female | calf | not pregnant | not lactating | Brevard | Atlantic Ocean | 37.05 | 39.15 | 151.21 | 31.34 |
| MEC10123 | 12/3/2010 | male | calf | not pregnant | not lactating | Indian River | Indian River | 18.91 | 43.40 | 76.84 | 19.36 |
| MEC10124 | 14/3/2010 | female | subadult | not pregnant | not lactating | Brevard | Banana River | 18.61 | 60.87 | 276.09 | 62.08 |
| MEC10125 | 13/3/2010 | male | calf | not pregnant | not lactating | Brevard | Indian River | 30.53 | 13.80 | 303.53 | 35.48 |
| MEC10126 | 13/3/2010 | male | calf | not pregnant | not lactating | Indian River | Indian River | 21.09 | 9.15 |  | 21.63 |
| MEC10128 | 15/3/2010 | male | subadult | not pregnant | not lactating | Brevard | Crane Creek | 9.16 | 28.68 | 370.92 | 92.30 |
| MEC10129 | 15/3/2010 | male | subadult | not pregnant | not lactating | Brevard | Sebastian River | 17.73 | 16.22 | 228.09 | 34.35 |
| MEC10131 | 16/3/2010 | male | subadult | not pregnant | not lactating | Brevard | Banana River | 5.41 | 14.92 |  | 27.59 |
| MEC10132 | 16/3/2010 | female | calf | not pregnant | not lactating | Brevard | Crane Creek | 22.61 | 35.87 |  | 136.01 |
| MEC10133 | 17/3/2010 | male | calf | not pregnant | not lactating | Brevard | Indian River | 12.16 | 69.43 |  |  |
| MEC10224 | 16/12/2010 | male | calf | not pregnant | not lactating | Brevard | Indian River | 14.54 | 23.03 | 2003.93 | 155.95 |
| MEC10227 | 19/12/2010 | female | calf | not pregnant | not lactating | Indian River | Sebastian Inlet | 5.90 | 14.12 |  | 95.71 |
| MEC10228 | 19/12/2010 | male | calf | not pregnant | not lactating | Brevard | Banana River | 28.52 | 15.44 | 563.53 | 66.76 |
| MEC10229 | 20/12/2010 | male | subadult | not pregnant | not lactating | Brevard | Indian River | 3.14 | 7.90 | 466.43 | 34.61 |
| MEC10230 | 20/12/2010 | male | adult | not pregnant | not lactating | Brevard | Indian River | 2.94 |  | 291.05 | 26.36 |
| MEC10231 | 20/12/2010 | female | calf | not pregnant | not lactating | Brevard | Sykes Creek | 8.85 | 20.17 |  | 64.91 |
| MEC10232 | 21/12/2010 | male | calf | not pregnant | not lactating | Brevard | Indian River | 13.91 | 18.42 | 223.56 | 26.68 |
| MEC10235 | 21/12/2010 | male | calf | not pregnant | not lactating | Brevard | Indian River | 20.11 | 68.91 | 592.31 | 179.79 |
| MEC10236 | 25/12/2010 | female | adult | not pregnant | not lactating | Brevard | Indian River | 4.86 |  | 649.80 | 62.36 |
| MEC10241 | 28/12/2010 | male | adult | not pregnant | not lactating | Brevard | Indian River | 15.10 | 30.29 |  | 149.26 |
| MEC10242 | 28/12/2010 | female | subadult | not pregnant | not lactating | Brevard | Barge Canal | 6.63 | 8.18 | 170.67 | 32.00 |
| MEC10243 | 29/12/2010 | female | calf | not pregnant | not lactating | Brevard | Indian River | 12.19 | 19.83 |  | 143.22 |
| MEC10245 | 30/12/2010 | female | calf | not pregnant | not lactating | Indian River | Sebastian River | 8.03 | 20.78 | 689.06 | 38.44 |
| MEC1092 | 19/2/2010 | male | calf | not pregnant | not lactating | Brevard | Sebastian Inlet | 11.17 | 34.59 | 261.45 | 74.35 |
| MEC1101 | 1/1/2011 | male | calf | not pregnant | not lactating | Brevard | Indian River | 10.98 | 27.99 | 236.18 | 59.77 |
| MEC1118 | 21/1/2011 | male | calf | not pregnant | not lactating | Brevard | Turkey Creek | 24.61 | 5.41 | 69.18 | 18.17 |
| MEC1126 | 29/1/2011 | male | subadult | not pregnant | not lactating | Indian River | Sebastian River | 10.20 | 12.53 | 117.59 | 20.12 |
| MNE1024 | 8/3/2010 | male | calf | not pregnant | not lactating | Volusia | Atlantic Ocean | 10.97 | 18.83 | 1078.76 | 82.28 |
| MNE1049 | 17/12/2010 | male | subadult | not pregnant | not lactating | St. Johns | Pablo Creek | 4.64 | 10.40 |  | 26.47 |
| MNE1050 | 28/12/2010 | male | adult | not pregnant | not lactating | St. Johns | Tolomato River | 3.58 | 12.70 | 244.33 | 38.27 |
| MNE1051 | 31/12/2010 | male | subadult | not pregnant | not lactating | Volusia | Norris Dead River | 4.87 | 17.55 |  | 211.87 |
| MNW1016 | 19/2/2010 | male | calf | not pregnant | not lactating | Manatee | Manatee River | 14.92 | 47.31 | 165.79 | 13.10 |
| MNW1068 | 21/12/2010 | female | calf | not pregnant | not lactating | Manatee | Tampa Bay | 20.39 | 16.00 | 522.98 | 64.00 |
| MNW1071 | 26/12/2010 | female | subadult | not pregnant | not lactating | Pinellas | Boca Ciega Bay | 17.03 | 18.51 | 751.88 | 88.10 |
| MNW1110 | 28/1/2011 | male | adult | not pregnant | not lactating | Pinellas | Klosterman Creek | 12.45 | 24.25 |  | 84.99 |
| MSE 1102 | 1/1/2011 | female | calf | not pregnant | not lactating | St. Lucie | Indian River |  | 39.66 | 1691.59 | 206.67 |
| MSE10111 | 21/12/2010 | male | calf | not pregnant | not lactating | Palm Beach | Lake Worth | 51.90 | 18.60 | 1001.60 | 106.83 |
| MSE10116 | 26/12/2010 | male | calf | not pregnant | not lactating | Okeechobee | Taylor Creek | 9.67 | 16.21 | 291.73 | 37.12 |
| MSE1055 | 19/2/2010 | male | subadult | not pregnant | not lactating | Martin | Indian River | 5.70 | 36.04 | 154.07 | 8.82 |
| MSE1063 | 6/3/2010 | male | calf | not pregnant | not lactating | St. Lucie | Indian River | 6.40 | 22.72 | 293.27 | 47.67 |
| MSE1064 | 7/3/2010 | female | calf | not pregnant | not lactating | Broward | Stranahan River | 13.62 | 31.05 | 213.02 | 25.27 |
| MSE1066 | 10/3/2010 | female | calf | not pregnant | not lactating | St. Lucie | Indian River | 17.02 | 40.23 | 245.82 | 48.94 |
| MSE1069 | 15/3/2010 | male | calf | not pregnant | not lactating | Broward | Middle River | 4.94 | 14.74 | 165.03 | 34.30 |
| MSE1070 | 16/3/2010 | female | calf | not pregnant | not lactating | Martin | St. Lucie River | 9.45 | 12.70 | 87.31 | 6.08 |
| MSE1101 | 31/12/2010 | male | calf | not pregnant | not lactating | St. Lucie | Indian River | 9.82 | 22.34 | 472.42 | 59.13 |
| MSE1106 | 11/1/2011 | male | calf | not pregnant | not lactating | St. Lucie | Indian River | 24.07 | 18.01 | 136.17 | 22.01 |
| MSTm1011 | 11/3/2010 | male | calf | not pregnant | not lactating | Indian River | Indian River | 15.09 | 15.09 | 79.49 | 15.05 |
| MSTm1101 | 7/1/2011 | female | adult | not pregnant | not lactating | Collier | Faka Union Canal | 12.37 | 6.13 | 93.19 | 8.46 |
| MSW 1123 | 19/1/2011 | male | adult | not pregnant | not lactating | Charlotte | Charlotte Harbor |  |  | 29.54 | 2.98 |
| MSW10138 | 18/2/2010 | female | calf | not pregnant | not lactating | Collier | Factory Bay | 10.42 | 21.21 | 4086.64 |  |
| MSW10140 | 20/2/2010 | female | calf | not pregnant | not lactating | Lee | Caloosahatchee River | 18.38 | 34.14 | 1141.80 | 310.17 |
| MSW10147 | 18/3/2010 | male | calf | not pregnant | not lactating | Collier | Gullivan Bay |  | 17.95 | 57.11 | 24.84 |
| MSW10155 | 1/3/2010 | female | calf | not pregnant | not lactating | Lee | Caloosahatchee River | 1.72 | 10.37 | 413.82 | 69.93 |
| MSW10156 | 5/3/2010 | female | calf | not pregnant | not lactating | Collier | Faka Union Canal | 49.18 | 22.40 | 106.93 | 33.72 |
| MSW10158 | 8/3/2010 | male | calf | not pregnant | not lactating | Collier | Faka Union Canal | 11.81 | 16.54 | 332.31 | 58.96 |
| MSW10161 | 16/3/2010 | male | calf | not pregnant | not lactating | Lee | Matlacha Pass | 17.48 | 14.12 |  | 45.15 |
| MSW10172 | 20/3/2010 | female | adult | not pregnant | lactating | Lee | Orange River |  | 8.07 | 147.29 | 22.15 |
| MSW10248 | 22/12/2010 | female | calf | not pregnant | not lactating | Lee | Ten Mile Canal | 15.14 | 15.87 | 129.49 | 2.12 |
| MSW10259 | 29/12/2010 | female | calf | not pregnant | not lactating | Charlotte | Lemon Bay | 10.76 | 4.69 | 200.06 | 31.21 |
| SWFTm1101b | 9/1/2011 | female | subadult | not pregnant | not lactating | Brevard | Indian River |  | 27.11 | 343.85 | 35.78 |
|  |  |  |  |  |  |  |  |  |  |  |  |

**Table A.3.** Descriptions and faecal cortisol (fC), aldosterone (fA), T3 (fT3) and T4 (fT4) metabolite concentrations (ng g^–1^) of wild Florida manatees that died of acute watercraft collision (WC) injuries. Missing hormone data was due to insufficient faecal sample sizes.

| **Manatee ID** | **Collection date** | **Sex** | **Size class** | **Pregnant** | **Lactating** | **County** | **Waterway** | **fC** | **fA** | **fT3** | **fT4** |
| --- | --- | --- | --- | --- | --- | --- | --- | --- | --- | --- | --- |
|  |  |  |  |  |  |  |  |  |  |  |  |
| *Acute WC* |  |  |  |  |  |  |  |  |  |  |  |
|  |  |  |  |  |  |  |  |  |  |  |  |
| MSE1054 | 19/2/2010 | female | adult | not pregnant | not lactating | Miami-Dade | Biscayne Bay | 5.06 |  | 122.92 | 30.83 |
| MNW1021 | 4/3/2010 | male | adult | not pregnant | not lactating | Hillsborough | Hillsborough Bay | 24.15 | 15.26 | 1389.63 | 122.47 |
| MSW10173 | 21/3/2010 | female | subadult | not pregnant | not lactating | Collier | Faka Union Canal | 40.46 | 11.27 | 240.50 | 32.76 |
| MEC10107 | 4/3/2010 | male | subadult | not pregnant | not lactating | Brevard | Indian River | 7.18 | 15.96 | 33.92 | 20.59 |
| MNW1061 | 11/11/2010 | male | subadult | not pregnant | not lactating | Franklin | Apalachicola Bay | 9.18 | 13.67 | 90.15 | 29.83 |
| MSE1104 | 8/1/2011 | female | adult | not pregnant | not lactating | Miami-Dade | Biscayne Bay | 4.92 | 20.90 | 53.15 | 37.93 |
| MSE1119 | 29/1/2011 | male | calf | not pregnant | not lactating | Palm Beach | Jupiter Sound | 3.55 | 14.60 | 304.69 | 36.11 |
| MSE1130 | 7/2/2011 | male | adult | not pregnant | not lactating | Broward | Hillsboro River | 3.69 | 9.31 | 244.66 | 35.98 |
| MSE10106 | 12/11/2010 | male | calf | not pregnant | not lactating | St. Lucie | Indian River | 4.10 | 10.37 | 93.47 | 11.51 |
| MSE10107 | 15/11/2010 | male | subadult | not pregnant | not lactating | Broward | Lettuce Lake | 1.97 | 4.45 | 101.51 | 14.50 |
| MSW1112 | 8/1/2011 | female | adult | not pregnant | not lactating | Lee | Estero Bay | 7.48 | 19.27 | 259.34 | 53.53 |
|  |  |  |  |  |  |  |  |  |  |  |  |
